# A healthy approach to dietary fats: understanding the science and taking action to reduce consumer confusion

**DOI:** 10.1186/s12937-017-0271-4

**Published:** 2017-08-30

**Authors:** Ann G. Liu, Nikki A. Ford, Frank B. Hu, Kathleen M. Zelman, Dariush Mozaffarian, Penny M. Kris-Etherton

**Affiliations:** 1 Freelance Medical Writer, West Lafayette, IN USA; 2 Director of Nutrition, Avocado Nutrition Center, Mission Viejo, CA USA; 3 Departments of Nutrition and Epidemiology, Harvard T.H. Chan School of Public Health, Boston, MA USA; 4 Director of Nutrition, WebMD, Marietta, GA USA; 5 Tufts Friedman School of Nutrition Science & Policy, Boston, MA USA; 6 Department of Nutritional Sciences, Pennsylvania State University, University Park, Pennsylvania, PA USA; 7 319 Chandlee Laboratory, Pennsylvania State University, University Park, Pennsylvania, PA 16802 USA

## Abstract

Consumers are often confused about nutrition research findings and recommendations. As content experts, it is essential that nutrition scientists communicate effectively. A case-study of the history of dietary fat science and recommendations is presented, summarizing presentations from an Experimental Biology Symposium that addressed techniques for effective scientific communication and used the scientific discourse of public understanding of dietary fats and health as an example of challenges in scientific communication. Decades of dietary recommendations have focused on balancing calorie intake and energy expenditure and decreasing fat. Reducing saturated fat has been a cornerstone of dietary recommendations for cardiovascular disease (CVD) risk reduction. However, evidence from observational studies and randomized clinical trials demonstrates that replacing saturated fat with carbohydrates, specifically refined, has no benefit on CVD risk, while substituting polyunsaturated fats for either saturated fat or carbohydrate reduces risk. A significant body of research supports the unique health benefits of dietary patterns and foods that contain plant and marine sources of unsaturated fats. Yet, after decades of focus on low-fat diets, many consumers, food manufacturers, and restauranteurs remain confused about the role of dietary fats on disease risk and sources of healthy fats. Shifting dietary recommendations to focus on food-based dietary patterns would facilitate translation to the public and potentially remedy widespread misperceptions about what constitutes a healthful dietary pattern.

## Introduction

The way consumers obtain nutrition information has changed substantially in the past two decades. Use of the internet and social media has grown rapidly, and these are now among the leading sources of information for health and wellness. Perhaps due to access to more information than ever, including conflicting information of uncertain and variable quality, many consumers are more confused than ever.

Nutrition scientists are trusted content experts [1]. Consequently, it is essential that they effectively communicate research findings to policy makers, authoritative bodies and the general public in order for consumers to make sound, evidence-based dietary decisions. In addition, communicating scientific findings can be viewed as a civic duty [2] and has been suggested to be included in formal academic training [2]. Yet, scientists’ ability to communicate is less than other professionals [1].

Within the nutrition community, one example of suboptimal communication between scientists and the public is the continued demonization and general avoidance of dietary fat [3]. For years, an emphasis of nutrition communication was to balance calorie intake and energy expenditure and decrease dietary fat. Reductions in total dietary fat were recommended to reduce saturated fat as well as due to the energy density of lipids and the overall goal to reduce caloric intake [4]. Partly as a result, low-fat, high-carbohydrate diets were recommended in 1980 and thereafter for weight loss and reducing cardiovascular disease (CVD) risk. However this led to unintended consequences. The focus on reducing total fat resulted in increased consumption of refined carbohydrates and added sugars, and avoidance of nutrient-dense foods rich in healthy unsaturated fats such as nuts, seeds, avocados and vegetable oils. Subsequently, fat consumption has decreased while carbohydrate intake has increased as percentage of calories, which has been accompanied by significant increases in total energy intake and obesity rates in the United States [5, 6].

While single nutrient targets have worked well for treating diseases of deficiency, this has been problematic for addressing chronic diseases [7]. In contrast, specific foods and overall dietary patterns can substantially affect chronic disease risk [8]. However, by attributing their effects to single nutrients, foods with very different physiological effects can become conflated and contribute to consumer confusion. A global survey found that 95% of respondents knew that vitamins were needed for a healthy diet, but only 41% knew certain fats were essential nutrients [9]. In recent decades, we have gained substantial knowledge regarding the role of broad classes of nutrients and foods in major chronic diseases. In addition, challenges in communicating science clearly to the public contributes to consumer confusion and, as a result, may have public health consequences. This paper summarizes an Experimental Biology Scientific Session for which the goals were to discuss the history of recommendations for dietary fat and evidence showing health benefits of unsaturated fats from plant sources in order to provide context for the strategies described to improve nutrition science communications to the public.

## Case study: Dietary fat

### History of dietary fat recommendations

Reducing dietary saturated fat has been a cornerstone of recommendations for reducing CVD risk for decades, largely based on the classic diet-heart hypothesis which proposes that dietary saturated fat and cholesterol play a primary role in the development of atherosclerosis and coronary heart disease (CHD). This hypothesis was informed by two key observations: 1) controlled feeding trials demonstrated that dietary saturated fatty acids and cholesterol raised serum total cholesterol and low density lipoprotein cholesterol (LDL-C) levels, and 2) increased serum total cholesterol and LDL-C predicted risk of CHD [10]. Since the origin of the diet-heart hypothesis, a large body of research has identified multiple pathways that mediate the development of CHD. Thus, interventions that affect single surrogate biomarkers must be interpreted with caution [11]. Though apolipoprotein B-carrying LDL-C particles are established causal determinants of CVD risk, there are many other CVD risk factors that substantially affect disease development including other blood lipids and lipoproteins, hypertension, smoking, diabetes, overweight and obesity. Dietary fats have complex and sometimes divergent effects on these different contributors to CVD risk. Though views of the original diet-heart hypothesis continue to evolve, they have had long-lasting effects on nutrition policy and consumer perceptions of fat. Decades of recommendations to consume low-fat diets and the proliferation of low-fat products have greatly influenced consumer perceptions of fat.

### Current recommendations for dietary fat intake

#### Total fat

The percentage of energy consumed as fat can vary widely, and the diet can still meet energy and nutrient needs. Current recommendations from various organizations regarding fat intake in adults are summarized in Table 1 [12–17]. Dietary guidelines from the World Health Organization and the Dietary Reference Intakes recommend a total fat intake between 20 and 35% of total calories [12, 13]. The minimum of 20% is to ensure adequate consumption of total energy, essential fatty acids, and fat-soluble vitamins [12] and prevent atherogenic dyslipidemia (low high-density lipoprotein cholesterol (HDL-C), high triglyceride-rich lipoproteins) which occurs with low-fat, high carbohydrate diets and increases risk of coronary heart disease [13]. The maximum of 35% was based on limiting saturated fat and also the observation that individuals on higher fat diets consume more calories, resulting in weight gain [13]. No Tolerable Upper Intake Level was set for total fat because there is no intake level for which there is an adverse event [13]. Of note, the 2015 Dietary Guidelines Advisory Committee placed emphasis on the types and quality of foods consumed and did not set an upper limit for total fat based on the lack of supporting evidence [14]. This was reflected in the Dietary Guidelines for Americans 2015–2020, which emphasizes types of fat within the context of a healthy dietary pattern [18].

**Table 1:** Current Dietary Fat Intake Recommendations for Adults

		Recommended Percent of Energy				
Organization	Report	Total	Saturated	Trans	n-6 PUFA	n-3 PUFA
World Health Organization	Fats and fatty acids in human nutrition: report of an expert consultation [12]	20–35%	<10%	<1%	2.5–9%	0.5–2%
Food and Nutrition Board, Institute of Medicine	Dietary reference intakes for energy, carbohydrate, fiber, fat, fatty acids, cholesterol, protein, and amino acids [13]	20–35%	Limit	Limit	5–10%	0.6–1.2%
United States Department of Health and Human Services and United States Department of Agriculture	Scientific Report of the 2015 Dietary Guidelines Advisory Committee [14]		<10%	Limit		
American Heart Association/ American College of Cardiology	Guideline on Lifestyle Management to Reduce Cardiovascular Risk, 2013 [15]		5–6%	Limit		
American Diabetes Association	Standards of Medical Care in Diabetes, 2015 [16]	Evidence suggests that there is not an ideal percentage of calories from carbohydrate, protein, and fat for all people with diabetes. Follow same recommendation as for the general population.				
American College of Cardiology/ American Heart Association/ The Obesity Society	Guideline for the Management of Overweight and Obesity, 2013 [17]	A variety of dietary approaches can produce weight loss in overweight and obese adults as long as reduction in energy intake is achieved. Weight loss is comparable with lower-fat and higher-fat diets.				

*PUFA* polyunsaturated fatty acids

#### Saturated fat

The 2015 Dietary Guidelines Advisory Committee, the Dietary Guidelines for Americans 2015–2020, and many other organizations consistently recommend a limitation on intake of saturated fat, typically to <10% of energy [12–15]. In contrast, Canada’s Heart and Stroke Foundation recently removed any specific limitation on saturated fat, stating instead that their dietary guidelines do “not include a threshold or limit for saturated fat and instead focus on a healthy balanced dietary pattern” [19]. The role of saturated fat for CHD, and the corresponding controversy, is discussed further below.

#### Trans fat

The Institute of Medicine determined that there is no safe level of consumption of industrial *trans* fats from partially hydrogenated oils. *Trans* fats adversely affect a diverse range of CVD risk factors: they raise LDL-C, raise triglycerides, lower HDL-C, increase inflammation, promote endothelial dysfunction, and may promote hepatic fat synthesis, resulting in far greater risk of developing CHD than any other macronutrient. Based on these effects, the recommendation is to limit their intake as much as possible [10]. Denmark was one of the first countries to ban the sale of products containing trans fats in 2003 and since that time the European Union has taken a stance to reduce trans-fats in the food supply [20] and, at the same time, the US Food and Drug Administration ruled in 2015 that partially hydrogenated oils are no longer Generally Recognized as Safe and should be removed from the food supply [21].

#### Monounsaturated fat

Like saturated fats, *cis*-monounsaturated fatty acids (MUFA) are readily synthesized by the liver in response to carbohydrate consumption [13]. They are not required in the diet; thus no Adequate Intake or Recommended Dietary Allowance has been set [13]. In addition, there is little evidence to set a Tolerable Upper Intake Level [13]. The major MUFA in Western diets is oleic acid which is abundant in both animal and plant sources [12]. Most dietary guidelines for MUFA consumption are based on subtraction of recommended intakes of saturated fat and polyunsaturated fat from total fat rather than evidence for specific optimal intakes of MUFA per se.

#### Polyunsaturated fat

*Cis*-polyunsaturated fatty acids (PUFA) include essential fatty acids and have beneficial roles in human health. However, formal clinical deficiency of n-6 and n-3 fatty acids is rare in healthy individuals in the United States and most other countries. More than a decade ago, the IOM set definitions of Adequate Intakes for linoleic and α-linolenic acid based on median US population intakes, with up to 10 % of the recommended total n-3 PUFA intake being eicosapentaenoic acid (EPA) and/or docosahexaenoic acid (DHA) [13]. These US Dietary Reference Intakes, based on evidence published prior to 2000, have not been updated. More recently, the United Nations Food and Agriculture Organization set new target Acceptable Macronutrient Distribution Ranges for adults for linoleic acid (2.5–9% of energy), total n-3 PUFA (0.5–2% of energy), and EPA + DHA (250 to 2000 mg/d) [12].

### Trends in consumption of dietary fats

Since 1971, the average fat intake in the United States has decreased from 36.6 to 33.6% [5]. The median intake of saturated fat currently is 9.7–11.1% depending on sex and race or ethnic subgroup, and approximately 42–65% of the adult population consumes greater than the recommended level of 10% of calories from saturated fat [22]. Since 1980, when the first Dietary Guidelines for Americans were issued, the intake of saturated fat has steadily decreased as a percent of calories. The decrease in total and saturated fat intake (as a %) since the 1980’s has largely reflected a corresponding increase in energy from dietary carbohydrate.

These dietary trends are not unique to the U.S. Data from the Australian Health Survey 2011–2013 show dietary trends mirroring those observed in the U.S. [23]. Additionally, a report by the USDA Economic Research Service compared food availability and dietary preferences and behavior between the U.S. and the European Union and concluded that the diets are more similar, than not and both the U.S and EU have reduced fat consumption over time [24].

### Role of saturated, monounsaturated, and polyunsaturated fat in coronary heart disease

Saturated and monounsaturated fatty acids are synthesized in the body for energetic, physiological, and structural functions, and they are present in many foods. For example, palmitic acid, the major saturated fatty acid in the diet, is synthesized in the liver from starch and sugar via de novo lipogenesis, and it is the predominant fatty acid present in dairy and meats [25]. Due to the positive linear relationship between total saturated fat intake and LDL-C concentrations, the recommendation is to limit saturated fat to <10% of calories [12–15]. However, the role of saturated fat in heart disease is complex because of the heterogeneous biological effects of the different saturated fatty acids and the diversity of food sources [26]. Moreover, conclusions are complicated by dietary substitutions underscoring the importance of considering the replacement nutrient.

Ecological and migration studies including the seminal Seven Countries Study by Ancel Keys have found strong positive correlations between saturated fat intake and CHD rates [10]. However, these studies are confounded by other environmental factors associated with different countries such as culture, geography, and economic development. Prospective cohort studies provide better evidence for dietary habits and CHD because adjustments are made for individual-level differences in major risk factors, lifestyle habits, and other confounding factors. While, these types of studies have consistently found that higher *trans* fat intake is associated with elevated risk of coronary heart disease [10], the effects of dietary saturated fat on coronary heart disease risk are less consistent [27]. A 2010 meta-analysis of prospective cohort studies by Siri-Tarino et al. found no relationship between total saturated fat and risk of coronary heart disease [28]. Similarly, a 2014 meta-analysis by Chowdhury et al. found no significant relationship between total saturated fat or total polyunsaturated fat consumption and risk of CHD [29]. These studies assessed the association of variations in saturated fat intake in the population, rather than modeling the specific substitution of saturated fat with other macronutrients. Studies specifically modeling the comparison of saturated fat to total carbohydrate have shown saturated fat to have similar associations with cardiovascular risk compared to total carbohydrate [30, 31]. Based on all the evidence, the 2015 Dietary Guidelines Advisory Committee concluded that replacing saturated fat with total carbohydrates does not reduce risk of CVD [32]. Recently, an AHA Presidential Advisory reviewed the scientific evidence and concluded that lowering intake of saturated fat and replacing it with unsaturated fats, especially polyunsaturated fats will lower the incidence of cardiovascular disease [33].

An alternative method for evaluating health effects of macronutrients is to consider the specific replacement nutrient. Such models do not compare differences in diet as actually consumed in the population, but provide estimates about potential health effects of specific inter-replacements of different macronutrients. In such models, the observed effects can be due to reduced intake of one nutrient, increased intake of the other, or both. Such models also raise complexities in understanding the biological effects of individual fatty acids within the context of food matrices and dietary patterns, which each provide a milieu of nutrients, bioactive compounds, and other constituents that may modulate the effects of the fatty acids.

**Fig. 1:**
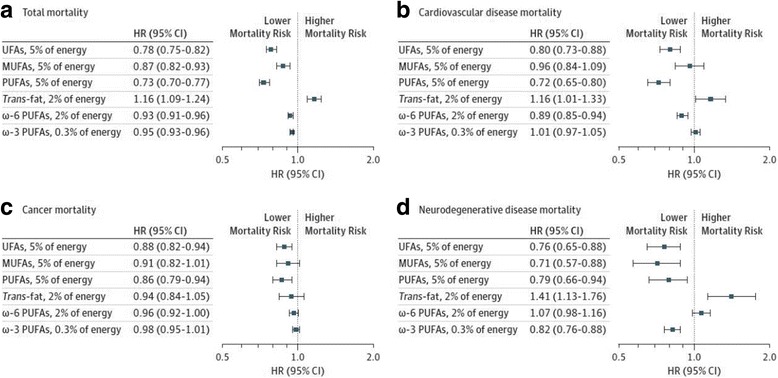
Effects of isocaloric substitution of specific fatty acids for saturated fatty acids in the Nurses’ Health Study and Health Professional Follow-up Study on **a**.) total mortality, **b**.) cardiovascular disease mortality, **c**.) cancer mortality, **d**.) neurodegenerative disease mortality. Results were from the multi-variate model using the fixed-effects model. UFA indicates unsaturated fatty acid and error bard, 95% confidence intervals. Reproduced with permission from Wang, et al. 2016 [35]

In cohort studies modeling specific replacement nutrients, there is consistent evidence that polyunsaturated fatty acids are the most beneficial replacement nutrient for CVD risk reduction as compared to either saturated fat or total carbohydrate. Jakobsen et al. pooled 11 cohort studies with over 344,000 participants and found that isocalorically replacing saturated fat with PUFA was associated with reduced risk of coronary events (per 5% energy, hazard ratio: 0.87; 95% CI: 0.77, 0.97) and coronary death (hazard ratio: 0.74; 95% CI: 0.61, 0.89) [34]. In a recent analysis, Li, et al. found that replacing saturated fat with high quality carbohydrates such as whole grains was associated with lower risk of CHD, but replacing saturated fat by total carbohydrates or refined starch/added sugars was not associated with CHD risk [31]. In contrast, other analyses, including a large pooling project that included the prior cohorts, suggest that total saturated fat is superior to total carbohydrate for CHD risk, and that refined starch/added sugars are more harmful than saturated fat. In the pooling project, isocalorically replacing saturated fat intake with either total carbohydrate or total MUFA did not result in reduced risk of coronary events; in fact, consuming total carbohydrate in place of saturated fat was associated with significantly higher risk (hazard ratio: 1.07; 95% CI: 1.01, 1.14) [34]. A recent publication of women from the Nurses’ Health Study and men from the Health Professionals Follow-up Study found that replacing carbohydrates with saturated fat was not associated with CHD mortality, while replacing carbohydrates with unsaturated fats significantly reduced CHD death [35]. Furthermore, substituting unsaturated fats for saturated fats (5% energy) reduced total mortality and mortality from CHD, cancer and neurodegenerative diseases (Fig. 1). A new study [36] reported that substituting plant protein for long chain SFA decreased risk of CHD. Another observational study found that replacing saturated fat with low glycemic index carbohydrates was associated with a nonsignificant trend toward lower risk of myocardial infarction, while replacement of saturated fat with high glycemic index carbohydrates was associated with significantly higher risk (hazard ratio: 1.33; 95% CI: 1.08, 1.64) [37]. The effects of replacing saturated fat with different types of carbohydrates require further investigation.

The similar associations of total carbohydrate vs. saturated fat with CHD (or in the largest studies, actually beneficial associations of saturated fat compared with total carbohydrate) might suggest that guidelines could include a limit on the sum of total carbohydrate plus saturated fat. The new research suggests that rather than focusing on total carbohydrate, the guidance should be on specific foods: limiting foods rich in refined starch and sugars, while eating more of other carbohydrate-containing foods such as fruits, legumes, and fiber-rich whole grains. Likewise, the new research suggests that rather than focusing on total saturated fat, the guidance also could be on specific foods, as saturated fat from different major food sources is associated with higher risk, no risk, or even lower risk of CHD, depending on the food source [38, 39]. For example, studies utilizing objective circulating biomarkers of fat intake identify protective associations of odd-chain saturated fats, largely consumed from dairy saturated fat, and risk of CHD [29]. These findings suggest that the specific matrix of different foods – including other fatty acids, nutrients, and bioactives – may biologically modify the effect of saturated fat on CHD. As is evident, this approach could be adopted for any single nutrient in the diet for providing food-based dietary guidance that also considers specific nutrient recommendations. Provocative new evidence suggests that we are at the beginning of a new era for making food-based dietary recommendations that requires more research and debate to reach scientific consensus.

When all these lines of evidence are considered, the role of saturated fat in CHD is controversial, including among the writing group of the present manuscript. Some scientists believe that reduction in saturated fat must continue to be prioritized, based on its LDL-raising effects and causality for CVD, on the benefits of replacing saturated fat with PUFA, and on concerns that in the absence of recommendations to limit saturated fat, ingredients high in saturated fat (e.g., palm oil) could be added to foods. Other scientists believe that heterogeneous effects of saturated fat on blood lipids and lipoproteins, of different individual saturated fatty acids, and of saturated fat from different food sources raises questions on the biologic and practical relevance of any focus on saturated fat, and that food-based recommendations are both more biologically sound and more practical.

Epidemiological evidence on the association of total dietary MUFA from all sources with CHD has been mixed [13, 34, 40]. A recent study, however, with Nurses’ Health Study and Health Professional Follow-up Study data estimated that replacing 5% of energy from saturated fat with MUFA was associated with a 15% lower risk of CHD [31]. In addition, another recent study has shown that replacement of saturated fat with MUFA (principally from plant sources) decreases CHD risk [36]. This observation likely reflects an increase in consumption of plant-based MUFA sources such as olive oil and a decreased consumption of animal-based MUFA sources from red meat over time, and thus the association for MUFA was less confounded by saturated fat. However, this finding needs to be confirmed in future studies.

**Fig. 2:**
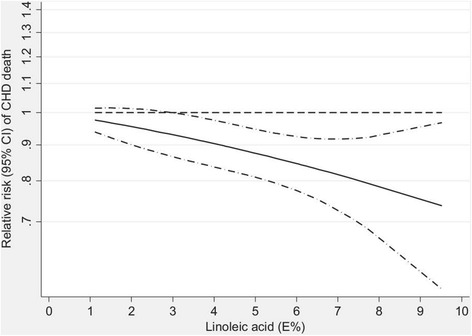
Dose–response analysis for the curvilinear association between dietary intake of linoleic acid and coronary heart disease deaths. *P* = 0.72 for nonlinearity relationship, indicating a linear relationship. %E indicates percent of energy. Reproduced with permission from Farvid et al. 2014 [41]

Many lines of evidence support CHD benefits PUFA consumption, whether as a replacement for saturated fat or carbohydrate. A meta-analysis of prospective cohort studies found that increased consumption of linoleic acid was associated with a 15% lower risk of CHD events (relative risk: 0.85; 95% CI: 0.78–0.92) and a 21% lower risk of CHD death (relative risk: 0.79; 95% CI: 0.71–0.89) [41]. The relationship was dose-responsive (Fig. 2) and independent of other traditional CHD risk factors and dietary factors such as fiber and α-linolenic acid [41]. Notably, benefits were similar irrespective of whether linoleic acid replaced saturated fat or total carbohydrate (which is often mostly refined).

**Fig. 3:**
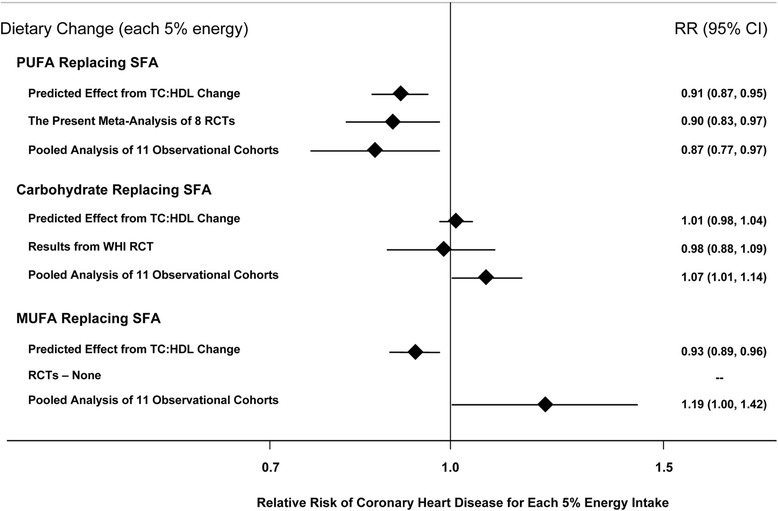
Effects on CHD risk of consuming PUFA, carbohydrate, or MUFA in place of saturated fat. Predicted effects are based on changes in the total cholesterol (TC):HDL-C ratio in short-term trials (e.g., each 5% energy of PUFA replacing saturated fat lowers TC:HDL-C ratio by 0.16) coupled with observed associations between the TC:HDL-C ratio and CHD outcomes in middle-aged adults (each 1 unit lower TC:HDL-C is associated with 44% lower risk of CHD) [42]. Evidence for effects of dietary changes on actual CHD events comes from the present meta-analysis of eight randomized controlled trials for PUFA replacing saturated fat and from the Women’s Health Initiative trial for carbohydrate replacing saturated fat (*n* = 48,835, ~3% energy reduction in saturated fat over 8 years) [81]. Evidence for observed relationships of usual dietary habits with CHD events comes from a pooled analysis of 11 prospective cohort studies [34]. Reproduced with permission from Mozaffarian et al. 2010 [30]

Evidence from clinical trials also supports the health benefits of increasing PUFA for reducing CHD risk. Mozaffarian et al. evaluated the effect of increased PUFA consumption, as a replacement for saturated fat, on CHD in a meta-analysis of randomized controlled trials [30]. Eight trials met inclusion criteria and encompassed 13,614 participants and 1042 coronary events (myocardial infarction or cardiac death) [30]. Average weighted PUFA consumption was 14.9% of energy in intervention groups and 5.0% in control groups. Increased PUFA consumption resulted in a 19% decrease in CHD risk (relative risk: 0.81; 95% CI: 0.70–0.95) [30]. Each 5% increase in energy from PUFA corresponded to a 10% decrease in CHD risk. Pooling across different types of evidence, consistent beneficial effects are seen when PUFA is increased, but not when SFA is replaced with carbohydrate or MUFA (Fig. 3) [30, 34, 42]. These studies provide compelling evidence that consumption of PUFA reduces CVD risk.

A growing body of literature suggests that both n-6 and n-3 fatty acids confer benefits for a wide range of conditions, in particular CVD, and also possibly diabetes, cancer, and autoimmune diseases [30, 41, 43–46]. The results of recent randomized controlled trials of n-3 PUFA supplements on cardiovascular outcomes have been disappointing; theorized reasons include the possibility that n-3 PUFA have little additional effect on top of modern drug therapies for CVD, as well as the study designs employed [47, 48]. However, these relatively short-term trials in high-risk populations may not be generalizable to the observed beneficial associations in generally healthy populations consuming dietary sources of n-3 PUFA such as fish [49]. Further research is needed to better determine how different approaches to food processing, technology, stability/oxidation, and breeding/engineering of plants or animals may alter the overall health effects of PUFA and MUFAs.

### Clinical interventions – Dietary patterns

Recent clinical evidence also supports the hypothesis that including plant and seafood sources of PUFA and MUFA in the diet improves cardiometabolic risk factors. Mediterranean diets generally derive a relatively high proportion of calories from fat (typically 35–40% of kcal or more) with much of the fat calories coming from plant and vegetable oils sources of MUFA [50]. Mediterranean-type diets commonly emphasize consumption of fruits, vegetables, legumes, fish, nuts, and olive oil [15]. In the Prevención con Dieta Mediterránea (PREDIMED) trial, 7447 persons were counseled to consume a Mediterranean diet supplemented with extra-virgin olive oil (50 g/day), a Mediterranean diet supplemented with mixed nuts (30 g/day; 15 g of walnuts and 7.5 g of almonds and 7.5 g of hazelnuts), or a control diet reduced in dietary fat [50]. After a mean follow-up of 4.8 years, consumption of a Mediterranean diet supplemented with either extra-virgin olive oil or nuts resulted in a 30% reduction in risk of myocardial infarction, stroke, or death (hazard ratio: 0.70; 95% CI: 0.54, 0.92 and hazard ratio: 0.72; 95% CI: 0.54, 0.96) [50].

Several secondary analyses of the PREDIMED trial have demonstrated other potential health benefits of consuming a Mediterranean diet. A Mediterranean diet supplemented with extra-virgin olive oil or nuts resulted in significant reductions in diastolic blood pressure, 24-h ambulatory blood pressure, fasting blood glucose, and total cholesterol [51, 52]. There were also reductions in biomarkers of vascular wall inflammation, which may partially explain the cardioprotective effects seen in the main study [53]. Participants who consumed a Mediterranean diet supplemented with extra-virgin olive oil or nuts also had a 52% reduction in diabetes incidence compared to the control group (hazard ratio: 0.47; 95% CI: 0.25, 0.97 and hazard ratio: 0.48; 95% CI: 0.24, 0.96) [54]. This suggests that changes in dietary patterns can have multifactorial health benefits beyond CVD.

The Dietary Approaches to Stop Hypertension (DASH) dietary pattern is also beneficial for reducing CVD risk. The original DASH diet emphasized vegetables, fruits, whole grains, low-fat dairy products, poultry, fish, and nuts while limiting sweets and red meats, and was generally higher in carbohydrates and lower in total fats. At the end of the eight-week dietary intervention, systolic and diastolic blood pressure were significantly reduced by 5.5 and 3.0 mmHg compared to the control diet [55]. Consumption of the DASH diet also resulted in lower total cholesterol, LDL-C, and HDL-C levels with no changes in triglycerides or total cholesterol:HDL-C ratio [56]. As a follow up to the DASH trial, the Optimal Macronutrient Intake Trial to Prevent Heart Disease (OmniHeart) was conducted to compare high-carbohydrate, high-protein, or high-MUFA versions of the original DASH diet. Participants with prehypertension or stage 1 hypertension were fed for 6-week periods in a 3-period randomized crossover trial. While all diets improved blood pressure and LDL compared to baseline, the diets that replaced saturated fat with protein or especially vegetable unsaturated fats (principally olive oil) resulted in greater improvements in CVD risk factors compared to the carbohydrate-rich diet [57].

These studies demonstrate consistent themes of dietary patterns that effectively reduce CVD risk. Accordingly, the 2015 Scientific Report of the Dietary Guidelines Advisory Committee concluded, “A healthy diet can be achieved in multiple ways and preferably with a wide variety of foods and beverages.” [14]. They also identified common features of beneficial dietary patterns across diverse health outcomes including cardiovascular disease, obesity, and cancer. The committee recommended healthy dietary pattern options that: 1) emphasize vegetables, fruits, whole grains, seafood, legumes, and nuts, 2) include moderate amounts of low-fat dairy products and alcohol (among adults, if consumed), 3) are lower in red and processed meats, 4) limit refined grains and sugar-sweetened foods and beverages [14].

### Clinical interventions – Specific foods and oils

Several studies have examined the potential benefits of incorporating specific foods and oils on cardiometabolic risk factors. As described above, PREDIMED demonstrated reductions in CVD events with either mixed nuts or extra-virgin olive oil. A systematic review and meta-analysis examined the relationship between nut consumption and blood lipid levels. A total of 61 trials (42 randomized, 18 non-randomized) totaling 2582 unique participants provided nuts to participants for durations ranging from 3 to 26 weeks [58]. Compared with controls, each daily serving of nuts lowered LDL-cholesterol (−4.8 mg/dL; 95% CI: -5.5, −4.2) [58]. These results complement previous findings from a pooled analysis of intervention trials examining the relationship between nut consumption and blood lipid levels [59]. Twenty-five trials comprising 583 participants were included. Interventions were at least 3 weeks in duration and nut consumption was the only dietary intervention [59]. Nut consumption (average 67 g/day) significantly reduced total cholesterol, LDL-C, and total cholesterol to HDL-C ratio [59]. Both studies are in agreement with the large body of epidemiological evidence showing an association between increased nut consumption and decreased risk of CHD [60, 61].

Olive oil is the main fat source in the Mediterranean diet, and it is believed to confer some of the cardioprotective benefits of the diet. Olive oil is high in MUFAs and contains phenolic compounds, which have antioxidant and anti-inflammatory properties [62]. Short-term clinical trials in healthy men have observed small increases in HDL-C, decreases in triglycerides, and reductions in systolic blood pressure with olive oil supplementation [62, 63]. Oxidative stress markers decreased with increasing polyphenol content of the olive oil [62]. These studies complement a previous observational study, which found an inverse association between olive oil consumption and both systolic and diastolic blood pressure [64]. In addition, the results are consistent with a study conducted with Nurses’ Health Study and Nurses’ Health Study II data that showed that substituting olive oil for stick margarine, butter, or mayonnaise was associated with a modestly lower risk of type 2 diabetes in women [65].

The Canola Oil Multi-center Intervention Trial (COMIT) sought to determine the effects of different oil blends with varying levels of n-9 MUFA, n-6 PUFA, and n-3 PUFA on biomarkers of coronary heart disease risk [66]. Participants were fed a controlled weight maintenance diet supplemented with one of 5 liquid vegetable oil treatments in a randomized crossover design. Treatments included: 1) conventional canola oil (Canola; n-9 rich), 2) high-oleic acid canola oil with docosahexaenoic acid (CanolaDHA; n-9 and n-3 rich), 3) a blend of corn and safflower oil (25:75) (CornSaff; n-6 rich), 4) a blend of flax and safflower oils (60:40) (FlaxSaff; n-6 and short-chain n-3 rich), or 5) high-oleic acid canola oil (CanolaOleic; highest in n-9). All treatments lowered total cholesterol and LDL-C [66]. The CanolaDHA blend significantly increased HDL-C, lowered triglycerides. The CanolaDHA blend had the greatest systolic and diastolic pressure-lowering effect. All treatments lowered the Framingham 10-year coronary heart disease risk score; the CanolaDHA treatment decreased it the most [66].

Foods high in plant sources of MUFA also have beneficial effects. A randomized crossover trial of the health benefits of daily avocado consumption was conducted with overweight or obese participants fed three cholesterol-lowering diets: 1) lower fat diet (24% fat), 2) moderate-fat diet (34% fat), and 3) moderate-fat diet supplemented with one avocado per day [67]; the latter two diets were matched for macronutrients. All three diets decreased LDL-C and total cholesterol compared to baseline [67]. The moderate-fat diet supplemented with avocado resulted in significantly greater reductions in LDL-C and total cholesterol than either the lower-fat or moderate-fat diet [67]. Additionally, the avocado-containing diet significantly reduced LDL particle number, small dense LDL, and the ratio of LDL-C/HDL-C [67]. These studies provide evidence of the lipid-lowering potential of plant foods that are rich in PUFA and MUFA. The results of this study also suggest additional benefits of nutrients/bioactives in avocados beyond their healthy fat composition.

**Fig. 4:**
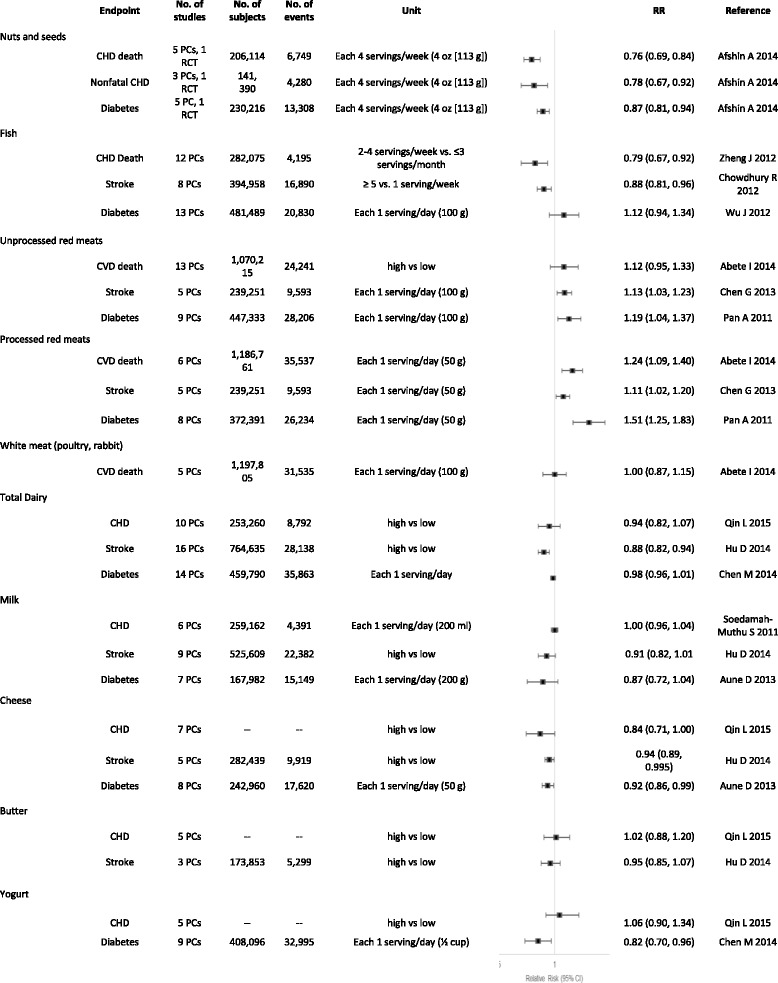
Meta-analyses of foods and coronary heart disease, stroke, and diabetes mellitus. BMI indicates body mass index; CHD, coronary heart disease; CI, confidence interval; CVD, cardiovascular disease; PC, prospective cohort; RCT, randomized clinical trial; and RR, relative risk. Adapted with permission from Circulation [74]

Interestingly, there have also been some studies suggesting possible cardioprotective benefits of certain foods high in saturated fats such as dark chocolate and specific dairy products. Meta-analyses of randomized controlled trials investigating the effects of chocolate or cocoa products have found inverse associations between cocoa consumption and total cholesterol, LDL-C, blood pressure, and serum insulin [68–70]. These effects have been attributed to the flavanols found in dark chocolate and cocoa products. Dairy products encompass a widely varied group of foods including butter, milk, cheese, and yogurt. Epidemiological studies have generally found no association or modest inverse associations between dairy product intake and risk of CVD [38, 71]. Clinical studies have been mixed depending on the dairy product and the comparator investigated. Butter and whole milk increase total cholesterol and LDL-C [72]. Cheese consumption seems to modestly lower LDL-C when compared to butter [72]. Yogurt consumption may produce favorable changes in LDL-C, HDL-C, and triglycerides, but the effects seem to be highly dependent on the strain of bacteria used for fermentation [72]. Prospective cohort studies indicate a consistent inverse association between yogurt consumption and risk of type 2 diabetes, although the association between other dairy products and diabetes risk has been inconsistent [73]. A recent compilation [74] of meta-analyses designed to evaluate associations between individual foods (and relevant to this paper, foods with different fat types) and coronary heart disease, stroke and diabetes is shown in Fig. 4. As is evident, nuts and seeds consistently show benefits, fish benefits CHD death and stroke but not diabetes, and dairy products, including total dairy, milk, cheese, butter, and yogurt demonstrate inconsistent associations. For processed red meats, there is clear evidence of increased associations with cardiometabolic diseases, whereas for unprocessed red meats, there is evidence for increased stroke and diabetes risk [74]. As noted in the paper, there is considerable controversy about cheese, low-fat milk and butter, as well as unprocessed red meat and their relationship to cardiometabolic health. Furthermore, for whole milk, there is insufficient evidence for meaningful conclusions.

### Public confusion about nutrition research and resultant dietary fat recommendations

As a case-study, the science on dietary fat and cardiovascular disease is complicated, therefore research communications and dietary recommendations should be made that accurately interpret the complexity of the evidence. When asked about information provided by governments, experts, food companies, and the media regarding the role of fats in a healthful diet, 64% of consumers were confused and felt that the information provided was contradictory [9]. The term fat is particularly confusing because 90% of survey respondents associate something negative with fat [9]. Most people, especially women, associate fat intake with obesity while older men are more likely to associate it with heart health [75]. In a recent poll of Americans, nearly 70% believed they should limit their fat intake to control their weight and reduce their risk of heart disease [76]. Survey results suggest that most consumers believe that their fat intake should be as low as possible and that fat is not needed for a healthy diet [9, 76]. Despite consumer perceptions, research supports the use of higher-fat diets such as Mediterranean-style diets for weight loss and reducing CVD risk [17, 50]. Indeed, excess consumption of calories has greater effects on weight and energy balance than the amount and type of fat consumed [17].

While the public is very aware of total dietary fat, they do not have a good understanding of the importance of fat quality or of the different sources of dietary fat. Pizza, grain-based desserts, and chicken and chicken mixed dishes are among the tops sources of various fats in the diet of the U.S. population as seen in Table 2 [77]. This reflects high levels of consumption of these items by consumers. When asked about whole food sources of fat, 3 out of 4 consumers identified olive oil and fish oil as being healthful [18]. However, only 1 in 2 consumers identified avocados and nuts as healthy source of fat [76]. When consumers were asked the same question using the terms monounsaturated fatty acids and polyunsaturated fatty acids, only 16% believed they were healthful, illustrating how the chemistry terms for categorizing fats do not resonate with consumers [18].

**Table 2:** Top food sources of different types of fatty acids in the diets of the U.S. population and recognized food sources

Type of Fat	Top Sources in the Diets of the U.S Population (Contribution to Intake)^a^	Food Sources
Saturated	Regular cheese (8.5%)	Cheese
	Pizza (5.9%)	Butter
	Grain-based desserts (5.8%)	Fatty cuts of meat
	Dairy desserts (5.6%)	Cream
	Chicken and chicken mixed dishes (5.5%)	Lard
	Sausage, franks, bacon, and ribs (4.9%)	Palm and coconut oils
	Burgers (4.4%)	
	Mexican mixed dishes (4.1%)	
Oleic acid	Grain-based desserts (8.9%)	Olive oil
(MUFA 18:1)	Chicken and chicken mixed dishes (7.6%)	Canola oil
	Sausage, franks, bacon, and ribs (5.9%)	Peanut oil
	Nuts/seeds and nut/seed mixed dishes (5.5%)	Avocados
	Pizza (5.4%)	Most nuts
	Fried white potatoes (4.9%)	
	Mexican mixed dishes (4.6%)	
	Burgers (4.1%)	
n-6 fatty acids	Chicken and chicken mixed dishes (9.5%)	Safflower oil
(PUFA 18:2 and 20:4)	Grain-based desserts (7.4%)	Sunflower oil
	Salad dressing (7.3%)	Soybean oil
	Potato/corn/other chips (6.9%)	Corn oil
	Nuts/seeds and nut/seed mixed dishes (6.4%)	Walnuts and walnut oil
	Pizza (5.3%)	
	Yeast breads (4.5%)	
	Fried white potatoes (3.5%)	
α-linolenic acid	Salad dressing (10.5%)	Flaxseeds and flaxseed oil
(PUFA 18:3)	Chicken and chicken mixed dishes (6.4%)	Canola oil
	Grain-based desserts (6.1%)	Soybean oil
	Pizza (5.8%)	Pumpkin seeds
	Yeast breads (5.0%)	Walnuts and walnut oil
	Mayonnaise (4.0%)	
	Pasta and pasta dishes (3.5%)	
	Quickbreads (3.4%)	
EPA and DHA	Other fish and fish mixed dishes (53.1%)	Salmon
(PUFA 20:5 and 22:6)	Chicken and chicken mixed dishes (13.8%)	Herring
	Shrimp and shrimp mixed dishes (12.9%)	Mackerel
	Eggs and egg mixed dishes (5.8%)	Anchovies
	Tuna and tuna mixed dishes (5.3%)	Sardines

^a^Based on data from National Health and Nutrition Examination Survey 2005–2006 and analysis by the National Cancer Institute [77, 87]

*EPA* eicosapentaenoic acid, *DHA* docosahexaenoic acid, *MUFA* monounsaturated fatty acids, *PUFA* polyunsaturated fatty acids

For prevention of chronic diseases, nutrient-based recommendations are more difficult to translate to the public. Few individuals can accurately estimate their daily calorie consumption, much less their intake of total fat or specific fatty acids [78, 79]. Interestingly, while 67% of consumers are trying to limit their fat intake, few are aware of how much fat they should actually be eating [76]. Only 22% of consumers correctly identified the recommended range of calories from fat [9]. Sixty percent of consumers believed that fat intake should be less than 14% of daily calories [9]. These results illustrate how single-nutrient-based targets can quickly become confusing to the average consumer. Based on the new science for benefits of fats, in particular healthful plant and seafood sources, and the harms of refined starches and added sugars, many scientists have called for the abandonment of the 35% limit on total fat which has been eliminated in the 2015 Dietary Guidelines (14) [80]. Consistent with this, the large Women’s Health Initiative trial demonstrated no benefits of lowering total fat from 36 to 29% of energy on risk for CVD, diabetes, or cancers; while the OmniHeart and PREDIMED trials demonstrated significant CVD and other benefits from increasing healthful fats to greater than 35% of energy [50, 57, 81–84]. Based on the scientific evidence, consumers should focus on overall dietary patterns and consume healthful foods rich in healthy fats including nuts, vegetable oils, other plant sources of fats, and substitute these for unhealthful foods such as processed meats and foods high in sodium, added sugars, or refined carbohydrates. This may result in a total fat intake that exceeds 35% of calories [80], but the majority of the fats in such a dietary pattern would be healthy fats. The 2015 Dietary Guidelines Advisory Committee strongly supported this shift toward focusing on foods and healthier dietary patterns, rather than individual nutrients or limits on total dietary fat [14, 85].

How should scientists communicate about fat in order to clear up the confusion? Simple, easily understood messages focused on overall dietary patterns and foods rather than single nutrients are important. As dietary guidance is shifting away from total fat reduction and instead emphasizing types of foods and overall dietary patterns, we should stop using low-fat terminology and instead talk about healthy foods. “How to” messages should inform the public of specific foods that are sources of “healthy fats”. Focusing on total diet quality and food patterns provides easily actionable messages for consumers rather than talking about percentages of specific fats.

Consumer confusion about nutrition messages can also result from conflicting headlines in the media, for example related to insufficient subject expertise by journalists; limited communication skills, availability, or willingness to be interviewed of nutrition scientists; or a need for eye-catching headlines in the fast-paced world of modern media. While it is crucial to present new studies in the context of the existing body of evidence, limited media space and consumer attention work against this. Indeed, new studies rarely negate previous findings or alter fundamental paradigms, but rather add new information to what was known before [86]. It is the responsibility of both scientists and the media to ensure that new results are accurately reported in appropriate context.

When communicating science, the following tips should be top of mind:

- Condense complex information into convincing and motivating messages, but keep them evidence-based.
- Use language at the 6th–8th grade reading level that is clear and easy to understand.
- The best messages are actionable, easy to implement, and easy to visualize.
- Remember to put research findings in context within the prevailing body of evidence and avoid sensational headlines [86].
- Work with reporters to make sure your comments and quotes are correct.
- Have a few (e.g. three) key messages that consumers can remember and reinforce with a strong bottom line.
- Specify practical dietary substitutions with a “compared to what” approach rather than general “eat more/less” [86].

In order to help the population achieve a healthy diet, communication will be needed on multiple levels including individual advice, media communication, and the development of programs and services at institutions such as schools, workplaces, and healthcare systems.

## Conclusions

Dietary fat is a confusing concept for the public, with both evolving science over time and areas of remaining uncertainty in the scientific literature. The resulting communication challenges are amplified by the complexities of evidence related to isolated nutrients vs. types of foods vs. overall dietary patterns. While each of these types of concepts can inform evidence-based nutrition science, and resulting dietary recommendations, they should not be considered in isolation without considering the overall types and quality of evidence. Indeed, reviewing the entirety of evidence allows the drawing of more valid conclusions regarding the health effects of certain classes of foods relative to other dietary choices.

We have presented evidence that the types of foods consumed and the overall dietary pattern followed are far more important for reducing CVD risk than total fat. Also the types of fat and carbohydrates – and more relevantly, the types of foods supplying these nutrients – are more important than the total amounts of fats and carbohydrates in the diet. Healthful plant and seafood sources of monounsaturated and polyunsaturated fats have important health benefits in the context of a healthy dietary pattern. Future dietary recommendations should focus on healthful dietary patterns to help consumers identify and choose foods that are good sources of healthy fats. Furthermore, dietary recommendations need to consider and incorporate principles for effective scientific communication as a top priority in order to effectively convey evidence-based scientific messages to the public.

## Abbreviations

CHD: Coronary heart disease
CVD: Cardiovascular disease
DHA: Docosahexaenoic acid
EPA: Eicosapentaenoic acid
HDL-C: High density lipoprotein cholesterol
LDL-C: Low density lipoprotein cholesterol
MUFA: Monounsaturated fatty acids
PUFA: Polyunsaturated fatty acids
